# Diabetology 4.0: Scoping Review of Novel Insights and Possibilities Offered by Digitalization

**DOI:** 10.2196/23475

**Published:** 2021-03-24

**Authors:** Claudia Eberle, Stefanie Stichling, Maxine Löhnert

**Affiliations:** 1 Medicine with Specialization in Internal Medicine and General Medicine Hochschule Fulda - University of Applied Sciences Fulda Germany

**Keywords:** diabetes mellitus, telemedicine, mobile apps, electronic health records, digital technology, eHealth, mobile phone

## Abstract

**Background:**

The increasing prevalence of diabetes mellitus and associated morbidity worldwide justifies the need to create new approaches and strategies for diabetes therapy. Therefore, the ongoing digitalization offers novel opportunities in this field.

**Objective:**

The aim of this study is to provide an updated overview of available technologies, possibilities, and novel insights into diabetes therapy 4.0.

**Methods:**

A scoping review was carried out, and a literature search was performed using electronic databases (MEDLINE [PubMed], Cochrane Library, Embase, CINAHL, and Web of Science). The results were categorized according to the type of technology presented.

**Results:**

Different types of technology (eg, glucose monitoring systems, insulin pens, insulin pumps, closed-loop systems, mobile health apps, telemedicine, and electronic medical records) may help to improve diabetes treatment. These improvements primarily affect glycemic control. However, they may also help in increasing the autonomy and quality of life of people who are diagnosed with diabetes mellitus.

**Conclusions:**

Diabetes technologies have developed rapidly over the last few years and offer novel insights into diabetes therapy and a chance to improve and individualize diabetes treatment. Challenges that need to be addressed in the following years relate to data security, interoperability, and the development of standards.

## Introduction

### Background

The ongoing digitalization changed the way of practicing medicine in general. Currently, it is possible to store and retrieve vast amounts of data and use different technologies to assist in diagnosis and therapy [1,2]. This technological development offers the opportunity to improve the treatment of diabetes mellitus (DM) in particular and in different ways [2,3].

In 2014, the global prevalence of DM among adults aged more than 18 years was estimated at approximately 8.5% and is believed to have increased since then, not taking into account the number of unreported cases [4]. As the high and rising prevalence and morbidity of DM, new strategies are needed.

In diabetes management, the measurement of blood glucose levels plays a key role. Therefore, different types of systems are available, which differ in when and how often the measurement takes place [5]. Furthermore, more than 200 million people with DM require insulin treatment [6]. Currently, there are mainly 2 possibilities to deliver insulin: multiple daily injections or continuous subcutaneous insulin infusions [5,6]. For both, different technological solutions are available in the market. Another important aspect of diabetes management depends on patient self-management. This means that people with DM must make several decisions, for example, when and what to eat, when to exercise, and, if required, the insulin dose and timing [7]. Technological innovations such as mobile health (mHealth) apps or advances in telemedicine may be used in different ways to overcome challenges such as the ones mentioned above [2,8-17]. The purpose of diabetes technologies is to avoid complications of insulin therapy and to give people with DM more autonomy so that their quality of life (QoL) can be improved [18]. Furthermore, it must be noted that we have considered all types of diabetes and that they have different therapeutic needs. Type 1 and type 2 diabetes have different pathogeneses, leading to various therapy recommendations and needs. In addition to some similarities, type 1 diabetes therapy focuses more on insulin therapy, whereas type 2 diabetes management focuses more on nutrition and exercise. In a closer analysis of the technologies, it is important to differentiate between the types of diabetes and their needs.

### Objective

The aim of this study is to provide an overview of the updated diabetes technologies available and the possibilities offered by digitalization of the treatment of DM.

## Methods

### Data Sources and Search Terms

We followed the PRISMA (Preferred Reporting Items for Systematic Reviews and Meta-Analyses) guidelines for scoping reviews to assess the scope of the literature on the topic [19]. The electronic databases MEDLINE (PubMed), Cochrane Library, Embase, CINAHL, and Web of Science were searched. For example, in PubMed, the following search strategy was used for a title and abstract search: if available in MeSH terms: “Diabetes Mellitus” OR “diabetes” OR “blood glucose assessment” AND “Telemedicine” OR “Mobile Applications” OR “digitization” OR “digital treatment,” an additional search in the reference lists and Google Scholar was conducted.

### Study Selection and Eligibility Criteria

The results of the literature search were transferred to the literature management program, Citavi, which was used to remove duplicates. Via a search of titles and abstracts, potentially relevant studies were extracted. Studies published in English or German between January 2008 and January 2020 that focused on technologies in DM therapy were included. Furthermore, to be included, the studies had to make remarks or comments on the clinical effectiveness, for example, effect on glycemic control, of the type of technology that was presented. There were no limitations to the study design. Studies that focused only on the comorbidities of DM were excluded. As this paper is a scoping review, we selected those studies from our presorted studies that seemed to give great overviews and explanations but still reported on clinical effectiveness.

### Data Extraction and Data Synthesis

The included studies were thematically categorized according to the type of technology presented. We then extracted the following data from each study: author, year, study design, objective, and main findings.

## Results

### Overview

Our search yielded a total of 4935 results, of which we included 67 studies (including systematic and narrative reviews). The search results can be categorized into the following types of technology: glucose monitoring systems, insulin pens, insulin pumps, closed-loop systems, mHealth apps, telemedicine, and electronic medical records (EMRs).

### Glucose Monitoring Systems

Most people with DM rely on self-monitoring of blood glucose (SMBG). Although there is a correlation between increased frequency of SMBG and a reduction in glycated hemoglobin A_1c_ (HbA_1c_), SMBG only gives a short impression of blood glucose levels without any information about the change in glucose levels and glucose variability [5]. To gain this information, continuous glucose monitoring (CGM) systems can be used.

CGM systems measure the glucose level at regular intervals, 24 hours a day, and translate the measurements into data about glucose direction and rate of change [18]. On the basis of this information, CGM use without additional SMBG is as safe and effective as CGM use with SMBG support [20]. In addition, several experimental studies have shown that the use of CGM systems improves glycemic control through a significant reduction in HbA_1c_ [21-25], and this has been shown in combination with insulin pump therapy [22] and with multiple daily insulin injections [24]. Another positive effect was demonstrated by Jones et al [26] in their systematic review targeting pregnant women with pre-existing diabetes, showing that a reduction in hypertensive disorders is possible when CGM is used. However, they assumed only low-quality evidence because only 2 studies included the outcome [26]. Further aspects of CGM can improve diabetes self-management, flexibility, and QoL. In particular, the option for alarms and the possibility of device connection with smartphones or smartwatches give the participants a feeling of greater safety and freedom [27]. Furthermore, CMG measures subcutaneous glucose as a substitute for blood sugar. Old systems had to be calibrated regularly, whereas newer systems did not require calibration, which benefits the patients.

The Juvenile Diabetes Research Foundation recognized an association between greater CGM use and a greater decrease in HbA_1c_ levels. However, they also noticed a decrease in CGM usage over the study duration, especially in children and adolescents [21]. Reasons for discontinuation include discomfort when wearing the CGM system, problems with inserting the sensor, or with the adhesive holding the sensor on the skin [28]. An alternative to minimize skin reactions is to use implantable CGM systems, which can be as safe and accurate as transcutaneous CGM systems [29,30].

Most of the studies relate to the so-called real-time CGM. Another type of CGM system is the intermittently scanned CGM system, also called flash glucose monitoring. These terms refer to CGM systems that can only be used on demand. This means that patients must use a reader device to scan the sensor to determine the glucose level [5]. This can be an alternative suitable for the masses because of lower costs relative to real-time CGM while also providing more information than with SMBG. However, it depends on the patients’ willingness to scan the device several times a day [31,32].

### Insulin Pens

Insulin pens are a technological invention for multiple daily insulin injections and are used as an alternative to insulin dosing via syringe. There are 2 types of insulin pens. Reusable pens have a delivery chamber in which insulin cartridges can be inserted. This makes it easy to change the type of insulin and is more economical than alternative prefilled pens. These contain a built-in single-use insulin cartridge. As a result, they are very convenient and easy to use, which is helpful for patients who have difficulties inserting the cartridges in reusable pens [33]. Overall, insulin pens have several advantages in terms of user friendliness, comfort of injections, and accuracy in delivering small doses of insulin. This leads to greater patient satisfaction and less reported pain [33,34].

However, there seems to be no improvement in glycemic control in insulin pens compared with the use of vials and syringes [35]. The advance in their development to so-called smart pens has changed this [36]. Smart pens include a memory function to track insulin doses and often have Bluetooth connectivity to transfer data to a smartphone app [18]. These functions seem to help patients improve their glycemic control [36].

The first FDA-approved smart pen, InPen, was launched in the United States in December 2017. In addition to digital diaries, it provides a dose calculator based on information on previous insulin doses, insulin on board, and current blood glucose levels. In addition, the app can be connected to some CGM systems [18,37,38]. This allows better diabetes management [39].

### Insulin Pumps

Insulin pumps are small programmable computerized devices that allow continuous subcutaneous insulin infusions, so a more physiological insulin release can be mimicked. This is realized by an individual basal insulin rate, which is released hourly and set by the medical staff. In addition, bolus insulin can be released after meals or as a correction to reduce the target glucose levels [40,41]. The use of insulin pumps is associated with a reduction in HbA_1c_ levels without an increased risk of hypoglycemia and seems to be more effective than multiple daily insulin injections [42-44].

There are different types of insulin pumps [40], and in a patch insulin pump, the insulin needle is a part of the pump and is inserted when the pump is attached directly to the surface of the skin. Controls of these pumps are on the device itself, or a remote can be used, so that there is no tubing. Examples are the V-Go and Omnipod insulin pumps [45,46]. Tethered insulin pumps have tubing between the pump itself and the cannula, so the pump can be worn under or outside clothing or carried around in a pocket. Examples are the Medtronic MiniMed 670G and Tandem t:slim X2 insulin pumps [47,48]. Although they are rarely used, other types are implantable insulin pumps. These are implanted into the peritoneal cavity and remain there; therefore, the users have to travel to get the pump refilled with insulin [40].

The current development in insulin pump technology is a direct and wireless communication between insulin pumps and CGM systems, for example, via infrared technology. Such systems are called sensor-augmented insulin pumps (SAPs) [41]. In contrast to closed-loop systems, SAPs still require manual adjustment and input from the wearer [49,50]. However, the use of SAP can further reduce the risk of hypoglycemic episodes and improve glycemic control [51-53]. SAPs can have different features; however, they often include a bolus calculator, an automated insulin suspension, and custom reminders. The bolus calculator calculates insulin doses for meals after the number of carbohydrates the wearer intents to eat are put in [49]. An automated insulin suspension allows the SAP to suspend insulin delivery when the CGM detects an unexpectedly low glucose level or risk of hypoglycemia [51]. Custom reminders can be set to remind the individual to check their glucose levels or to set a bolus amount. In addition, safety alerts for missed bolus doses or blockage in the tubing or cannula can be set [49,54].

### Closed-Loop Systems

Closed-loop systems, also referred to as artificial pancreas, are a combination of a CGM system and an insulin pump that can release insulin automatically [50]. These systems can be understood as fully automatic pumps that replace an endocrine pancreas; however, there are currently no such pumps in the market. Closed-loop pumps need to be calibrated, and patients need to assess their carbohydrate intake and tell the pump the time and amount of carbohydrates they will eat.

A step between SAPs and closed-loop systems are hybrid closed-loop systems such as MiniMed 670 G. This means that next to the manual mode where a basal insulin rate is set, they have the ability to use an automatic mode where insulin delivery is adjusted based on the CGM results. However, meal-related boluses remain user-dependent based on their carbohydrate intake [47,55,56]. Nevertheless, hybrid closed-loop systems have shown improved HbA_1c_ levels and time in range and decreased time spent in hypo- and hyperglycemias [55,57,58]. In addition, a closed-loop system consisting of the t:slim X2 insulin pump, Control-IQ Technology, and Dexcom G6 as a CGM system has been shown to improve glycemic control and time in range [59,60].

In addition, the #WeAreNotWaiting movement has developed systems for a do-it-yourself artificial pancreas (DIY AP). They used CGM systems, insulin pumps, and smartphone technology to run algorithms to calculate the insulin dose. These algorithms are openly shared by the community and have introduced various systems consisting of different components and using different interfaces. It must be noted that DIY AP are unregulated and therefore have potential safety concerns [61]. Although there are mainly observational studies about the effectiveness of DIY AP, they seem to improve the HbA_1c_ levels [62,63].

### Telemedicine

Despite the advances in glucose monitoring systems and improvements in insulin delivery, the proportion of people with DM achieving their glycemic control targets has not improved [64]. As a key contributor to poor glycemic control, *therapeutic inertia* is considered. *Therapeutic inertia* is defined as failure of initiation or intensification of therapy in a timely manner according to evidence-based clinical guidelines. It is a multifactorial problem, in which different stakeholders such as patients, health care providers, or the industry are involved [65,66]. Moreover, it underlines the need for care between visits to health care providers in diabetes treatment [67]. Telemedicine is becoming increasingly important as a solution to this problem [66]. Telemedicine describes the use of telecommunication systems to deliver health care at a distance, for example, through remote monitoring or real-time video conferencing [68]. This means that patients can communicate with their providers in an efficient way. However, health care providers can quickly provide feedback [67].

The open definition of telemedicine provides scope for various interventions, such as phone coaching interventions [69], tele-education [70,71], teleconsultations [72], real-time transmission of blood glucose levels [73], and web-based case management programs [74]. In addition, a combination of different components is possible [68,75]. Although there is a lack of evidence on long-term health outcomes, it is estimated that telemedical interventions lower HbA_1c_ levels with high certainty [68,76-79]. In addition, improvement in blood glucose and cholesterol, QoL, and the number of SMBG checks is possible [77,78]. This can be especially important in rural and medically underserved areas [71].

### mHealth Apps

Apps for mobile devices such as smartphones focusing on health-related topics are called mHealth apps, and as the app market is very dynamic, they exist in a large variety. Diabetes apps vary in their range of considered topics and features [9,10,12,13,16,17,80-82]. Common features include tracking of blood glucose levels or insulin usage, calculating an insulin dose, monitoring of diet, weight or physical activity, providing education, or allowing communication and exchange of data with health care professionals or social networks [2,8-17,80-84].

Even with these features, there is great variability and development [16]. For example, attempts have been made to create bolus calculators that provide personalized and adaptive insulin recommendations [85] or to create apps that use augmented reality to improve estimations regarding carbohydrate intake [86]. With such features, apps support diabetes self-management, especially for people using SMBG [5,84,87-89]. In addition, some apps are able to connect with other devices such as CGM systems, smart pens, or insulin pumps. Some devices come with their own app, for example, apps belonging to CGM systems allow wireless synchronization between the glucometer itself and an individual’s smartphone [81]. However, even with these features, not all needs expressed by people with DM are met. Adults with type 2 DM additionally wish for features that help in reducing the cognitive and emotional burden of diabetes self-management [90]. This is in line with recent findings showing that apps fail to provide relevant features to empower patients, even though there were complaints about a lack of personalized feedback, lack of education provided, and usability issues in the past [83,87,91].

Features such as writing a digital diary instead of a standard paper diary are not considered to affect QoL [92]. However, because of the combination of different features in most apps, it is difficult to determine the impact of specific app features on clinical outcomes [8]. Diabetes apps are supposed to improve glycemic control through significant reduction of HbA_1c_, although evidence on safety and effectiveness is limited, especially regarding long-term outcomes [93-95].

Nevertheless, mHealth apps have several advantages. Nearly all patients and health care professionals have smartphones, and in contrast to diabetes devices, which are often left behind, smartphones are almost always in a patient’s reach [81,96]. Moreover, apps have the ability to display data in colored charts and tables, which can be updated live and transferred via email or in cloud-based web portals [81]. In addition, an improvement in the reliability of patients’ diabetes data is possible because errors such as illegible handwriting or incomplete data entries can be minimized [81,97]. However, some disadvantages should be considered before using or recommending apps. First, there is a risk that during constant updates, bugs emerge and features are removed without warning or new pricing models are introduced [81]. Furthermore, especially, older patients can have special needs, such as a large font size because of bad eyesight, so not all apps suit them [98]. Moreover, apps are mostly not regulated by a governing body unless they meet the definition of medical devices. This holds a potential safety risk, especially regarding possible misinformation and data security [2,81,94]. Finally, it must be considered that some apps are only available on one operating system, Android or iOS [81]. This means that individualized recommendations should be provided to people with DM.

### EMR

The use of EMR offers the opportunity to transfer data of diabetes technology, such as glucose monitoring systems, insulin pumps, or insulin pens, directly to the patients’ medical records. This makes everyday life in primary health care easier and is a step toward an automated institution. This can be realized by setting up stations where patients can transfer their data to their EMRs. To achieve this service for devices of different providers, collaboration with engineers and technicians is needed [99]. Another possibility is the use of apps to transfer data into the EMR. The prerequisite for this is that the systems used are compatible with one another, for example, the platform *HealthKit* of the mobile device company Apple can be used with compatible CGM systems and compatible EMR vendor patient apps [100].

In addition to their use in primary health care, the collected data of EMRs can be used in public health surveillance to better understand the disease [101]. However, at present, there is a lack of quality in the collected data and more structured variables are needed to exploit the full potential of this use [102].

## Discussion

### Principal Findings

In total, digital diabetology has a huge potential to improve the health and QoL of patients with DM, especially glycemic control and diabetes therapy, using glucose monitoring systems, insulin pens, insulin pumps, closed-loop systems, mHealth apps, telemedicine, and EMR.

The variability of diabetes technologies shows that in the past decades, there has been a rapid development of care-supporting technologies. Advances in reducing the size and improving the accuracy of devices are not yet mentioned. It is believed that in the next 5 years, there will be many more advances, especially in CGM systems and automated insulin delivery through closed-loop systems. This includes the expectation that more systems will be available in the market [103]. Nevertheless, there are some points about the technologies that need to be discussed.

The advances in all the aforementioned technologies seem to improve glycemic control and, consequently, QoL. Therefore, they fulfill the purpose of diabetes technologies [18]. However, more data, especially on long-term outcomes, are needed [8,25,42,76,95]. Moreover, the technologies differ in the kind of improvement they make and their effectiveness. For example, insulin pens are an improvement compared with syringes in terms of pain and user friendliness; however, improvement in glycemic control seems to be possible with smart pens [33-36]. In contrast, insulin pumps are more likely to improve glycemic control than insulin pens [42-44]. However, insulin pump therapy does not suit everybody; for example, people doing athletics could experience problems with their pump because of failure or disconnection [39,104]. Therefore, an individualized solution that considers the patients’ needs and expectations is needed. This applies, in particular, to the recommendation of a diabetes mHealth app. It is possible that younger patients are more likely to benefit from an mHealth app intervention or that older patients have special needs that must be considered [93,98]. In addition, factors such as patients’ digital literacy should be considered. This is important because, despite the widespread use of smartphones, digital literacy barriers are common in vulnerable populations, which could reduce the effectiveness of diabetes technologies [105].

For such considerations, well-trained and educated health care personnel are needed [106]. Diabetes technologies can save time for patients who would spend less time at health care institutions and for health care personnel, because many processes can be automated [72,106]. However, this is still a type of future vision, because often enough missing interoperability between devices, especially of different providers, prohibits the automation of processes [107]. This goes hand in hand with missing standards and regulation of, for example, mHealth apps, which lead to potential health risks, for example, through misinformation [81,94,106]. In addition, the question of data safety is a major issue. Because these technologies deal with sensible patient data, protection must be of the highest priority. However, the missing regulation, specifically in mHealth apps, can lead to providers who store user data on remote servers that are more vulnerable to security breaches [81,94,106,107]. Therefore, there are still some aspects to address in the future.

### Limitations

It should be noted that this study is a scoping review. This means that the presented data are not complete and provide an updated overview of the area of digital diabetology. Systematic reviews and meta-analyses should be taken into account to investigate the effects of technology type on diabetes therapy in more detail.

The technologies themselves also have limitations that must be considered. There is a time lag in tissue-glucose results and insulin effects, which affects digital therapy approaches. In addition, there must be a certain willingness on the part of the patient to use the technologies and the patient requires skills to use the tools.

Furthermore, some studies showed rather small intervention effects with regard to HbA_1c_ levels. The effect sizes must be examined in more detail.

### Conclusions

In general, the different types of diabetes technologies offer a chance to individualize diabetology and improve the health and QoL of people with DM. With a look at the great advances that have been made in the last few years, rapid further development of diabetes technologies can be expected. In particular, the opportunity to connect different devices to create an automated insulin delivery will become more important. However, to use the full potential of digitalization, regulations and laws must be created to set standards and ensure data security.

## Abbreviations

CGM: continuous glucose monitoring
DIY AP: do-it-yourself artificial pancreas
DM: diabetes mellitus
EMR: electronic medical record
HbA_1c_: glycated hemoglobin A_1c_
mHealth: mobile health
QoL: quality of life
SAP: sensor-augmented insulin pump
SMBG: self-monitoring of blood glucose
